# The Stability of Refined Rapeseed Oil Fortified by Cold-Pressed and Essential Black Cumin Oils under a Heating Treatment

**DOI:** 10.3390/molecules27082461

**Published:** 2022-04-11

**Authors:** Monika Fedko, Dominik Kmiecik, Aleksander Siger, Małgorzata Majcher

**Affiliations:** 1 Department of Gastronomy Science and Functional Food, Faculty of Food Science and Nutrition, Poznan University of Life Sciences, Wojska Polskiego 31, 60-634 Poznan, Poland; 2 Department of Food Technology of Plant Origin, Faculty of Food Science and Nutrition, Poznan University of Life Sciences, Wojska Polskiego 31, 60-624 Poznan, Poland; dominik.kmiecik@up.poznan.pl (D.K.); malgorzata.majcher@up.poznan.pl (M.M.); 3 Department of Food Biochemistry and Analysis, Faculty of Food Science and Nutrition, Poznan University of Life Sciences, Wojska Polskiego 31, 60-634 Poznan, Poland; aleksander.siger@up.poznan.pl

**Keywords:** heating oil, triacyloglicerols polymers, polar compounds, tocochromanols, tocopherols, antioxidants, cold-pressed oil, essential oil

## Abstract

Polar compounds and polymers are regarded as the most reliable indicators of oil degradation during heating, and it is desirable to find methods to reduce these undesirable changes. The aim of this study was (1) to determine the effect of enrichment with black cumin cold-pressed oil (CP) or essential oil obtained from black cumin cold-pressed oil in an equivalent amount (ES) on limiting the polar compounds and polymers content in blends based on refined rapeseed oil during high-temperature heating in a thin layer; (2) to determine tocochromanol losses and their effect on the change content of the polar compounds and polymers. Four fortified oils were made from refined rapeseed oil and one of the four additives (10% CP, 20% CP, 0.1% ES, and 0.2% ES). All fortified oils and refined rapeseed oil as a control sample were heated at 170 and 200 °C on the pan in a thin layer and evaluated regarding loss of individual tocochromanol homologs by HPLC-FL, polar compounds content, oxidized triacylglycerols (TAG), and polymers content by HPSEC-ELSD. Additionally, the fatty acid profile in nonheated oil was investigated. Tocochromanol analysis showed loss in all the samples. At 170 °C polymers were not detected; no difference was noted for polar compounds and oxidized TAG content; only the 20% CP sample showed a higher level. At 200 °C the 10% CP sample exhibited a significant protective effect with the lowest content of polar compounds, oxidized TAG, and dimers.

## 1. Introduction

Pan frying is one of the world’s most widespread food preparation techniques. Dishes prepared in this way have sensory characteristics of taste, aroma, crunchy texture, and appearance that are much desired by consumers. Moreover, this method allows one to prepare a meal in a quick, uncomplicated, and relatively inexpensive way, and it can be applied with commonly available ingredients and tools.

On the other hand, high temperature and oxygen access during frying lead to many complex changes in the oil. Mainly, this consists in a degradation of triacylglycerols (TAG) and minor components like tocochromanols. These reactions generate products including oxidized TAG monomers, oxidized and non-oxidized TAG polymers, aldehydes, and ketones. All these substances may be transported into the frying products together with the oil. It has been shown that oxidized oil degradation products increase oxidative stress in the digestive system [1] and thus increase the risk of cancer and inflammation. Many studies also indicate a possible relationship between the consumption of lipid oxidation products and the occurrence of health problems such as disorders of the liver and of lipid and glucose metabolism, as well as atherosclerosis, hypertension, and Alzheimer’s and Parkinson’s diseases [2,3,4]. It was also found that a diet with increased content of polar compounds and TAG polymers had a harmful effect on the blood lipid profile and caused liver damage in laboratory rats [5,6]. Moreover, it is known that increased polymers content raises oil viscosity [7,8]. It leads to excess oil adsorption by the food and thus increases the caloric value of a dish [9]. In addition, numerous studies show that the consumption of fried food may contribute to other health problems, including cardiovascular diseases, diabetes, metabolic syndrome, overweight, and obesity [10,11,12,13]. All these results show that it is necessary to reduce oil degradation products in fried food. Hence synthetic antioxidants such as TBHQ, BHT, or BHA have been added to oils for over 70 years. However, these substances currently tend to be abandoned due to their toxic and carcinogenic effects [14]. For these reasons, a desirable direction of research is the search for natural additives limiting the thermal degradation of oils. Our previous study has shown that good results can be obtained by adding even small amounts of plant extracts containing antioxidants such as polyphenols [15]. The problem consists in the polar nature of these compounds. This causes low oil solubility and thus reduces practical possibilities of use. Cold-pressing is a technology of producing oil from seeds without heat or chemicals, and thus it retains valuable phytochemicals. Cold-pressed oils, which can be easily mixed with refined oils used for frying, are a rich source of native, lipophilic, antioxidant, and anti-polymerization biocomponents, such as tocochromanols, phytosterols, phenolic compounds, and volatile components [16,17,18]. So far, only a few types of cold-pressed oils have been tested for the stability of their blends with refined oils at high temperatures. Cold-pressed black cumin (*Nigella sativa*) oil is rich in bioactive substances including tocopherols and tocotrienols [19,20]. Moreover, black cumin seeds contain essential oil, in which one of the main components is thymoquinone [20,21]. According to various studies, both cold-pressed and essential black cumin oils have antioxidant properties [22,23], but their protection effect during heating treatment has not been known so far. It should be considered that the addition of black cumin seed oil to rapeseed oil will increase the proportion of polyunsaturated fatty acids (PUFA) in the fatty acid profile, which may adversely affect the thermal stability of the final blend. The essential oil addition would introduce some phytochemicals presented in cold-pressed oil without a change to the fatty acids profile. Furthermore, essential oil is easily mixed with fats, and essential black cumin oil can be obtained directly from seeds but also from fixed oil. An interesting avenue for research would be the evaluation of black cumin cold-pressed oil and essential oil obtained from it in an equivalent amount as enriching additives in oil and comparing their effect on limiting oil degradation during heating.

Considering all the arguments mentioned above, an innovative direction of research would be to use cold-pressed black cumin oil and essential black cumin oil as oil-soluble additives containing plenty of bioactive components with a potential for reducing the deterioration of oils during heating.

The aim of our research was (1) to determine the effect of enrichment with black cumin cold-pressed oil (CP) or essential oil obtained from black cumin cold-pressed oil in an equivalent amount (ES) on limiting the polar compounds and polymers content in blends based on refined rapeseed oil during high-temperature heating in a thin layer, and (2) to determine tocochromanol losses and their effect on the change in content of polar compounds and polymers.

## 2. Result and Discussion

### 2.1. Material Characterization

#### 2.1.1. Black Cumin Essential Oil

GC–MS analyses found fifteen compounds in the black cumin essential oil which are listed in Table 1. The major components were p-cymene, thymoquinone, and α-thujene, which together constituted 72.38% of the total oil. Other authors [21,24,25,26,27] reported a very diverse composition of black cumin essential oil. p-cymene content amounts to 7.07–59.5%, thymoquinone to 0.5–76.7%, α-thujene to 0.3–18.93%, carvacrol to 0.55–10.77%, and thymol from trace amounts to 10.12%. The essential oil composition may depend on many factors, including the method of obtaining the oil or species seeds as well as season, conditions, and place of cultivation.

#### 2.1.2. The Fatty Acids Profile and Iodine Value in Blends and Refined Rapeseed Oil

The results of fatty acid composition and iodine value of nonheated refined rapeseed oil and fortified oils are given in Table 2. The major components of the oils were C18:1 (65.60–57.69%), followed by C18:2 (25.91–18.46%) and C18:3 n-3 (8.57–7.18%). Four fatty acids palmitoleic (C16:1), arachidic (C20:0), behenic (C22:0), and erucic (22:1) were detected in amounts less than 1%. These fatty acids are typically for rapeseed and black cumin oils [28,29]. As may be expected, refined rapeseed oil and oils fortified by essential oils showed similar fatty acid composition and were characterized by a high share of monounsaturated fatty acid (MUFA), a low share of polyunsaturated fatty acid (PUFA), and low iodine value. In turn, with the increasing content of cold-pressed black cumin oil in the mixture, the MUFA share decreased while the PUFA share and iodine value increased.

#### 2.1.3. Tocochromanols in Blends and Refined Rapeseed Oil

Tocochromanol composition is presented in Table 3. α-, β-, γ-, δ-tocopherols, and plastochromanol-8 (PC-8) were identified in all of the analyzed oils. Additionally, α- and β-tocotrienols were detected only in 10% CP and 20% CP samples, and they are typical for black cumin oil [30]. γ-tocopherol was the most abundant tocochromanol in all raw blends (33.65–27.67%), closely followed by α-tocopherol (29.26–23.78%). Next, PC-8 was found with the content of 4.62–3.81%. α- and b-tocotrienols occurred in 10% CP with a content of 0.55–1.12% and in 20% CP with a content of 0.78–2.72%. Only a minor amount of β- and δ-tocopherols were found in all oil (below 1%). Total tocochromanol content ranged from 59.65 to 68.35%. The composition of RwA is comparable with the results for refined rapeseed oil described in other publications [31,32].

### 2.2. Heated Blends and Their Deterioration

#### 2.2.1. Tocochromanol Loss

**Figure 1:**
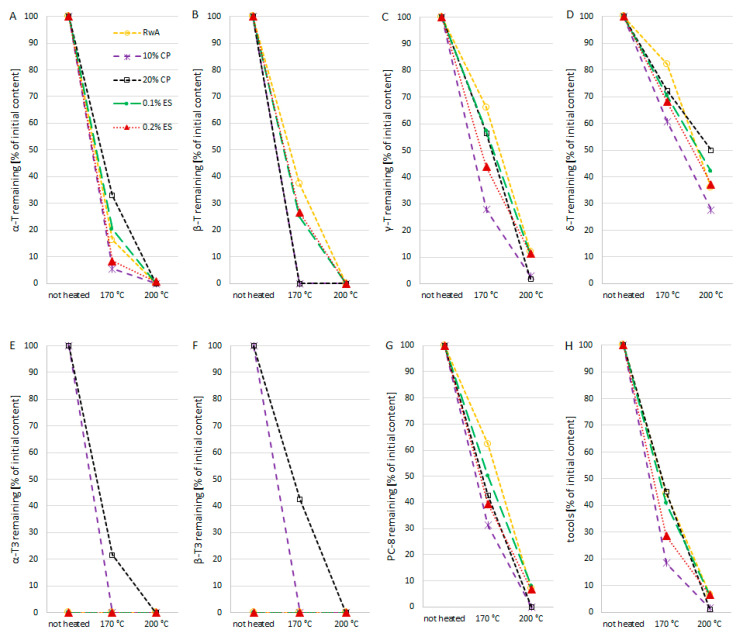
Changes of tocochromanols content in refined rapeseed oil and its blends during thin-layer heating at 170 and 200 °C. Values are means of four determinations. T—tocopherols (**A**–**D**); T3—tocotrienols (**E**,**F**); PC-8—plastochromanol-8 (**G**); total tocochromanols (**H**).

The heating process led to the loss of tocochromanols and in some cases their complete degradation (Figure 1). Among oils heated at 170 °C, the lowest remaining level of tocochromanols was characterized by 10% CP samples (18.48% of initial content). Coincident observation was made for α-, γ-tocopherols, and PC-8 in this sample (5.55, 27.86, and 31.32% of initial content, respectively). The remaining tocochromanols in 0.1% ES and 20% CP reached similar levels: 40.82% and 45.07%, respectively. Finally, the highest remaining tocochromanols in 170 °C were observed for 20% CP (45.07%). In turn, in all oils heated at 200 °C remaining tocochromanols were below 7% of the initial content. Such considerable losses in remaining tocochromanols were related to the significant share of α-tocopherol in nonheated oil, which was noticeably reduced during heating. In oils heated at 170 °C, its content ranged from 5.55 to 32.99% of the initial value and 200 °C from 0.72% of the initial value to an undetected level. γ-tocopherol and PC-8 were more stable, and δ-tocopherol turned out to be the most stable, with a content of 54.71–72.19% and 27.62–49.86% of the initial value in oils heated at 170 and 200 °C, respectively. Among oils heated at 170°, β-tocopherol was found only in RwA, 0.1% ES, and 0.2% ES, and α- and β-tocotrienol were found only in 20% CP. However, in oil heated at 200 °C they were not detected in any oils. These data are consistent with previous studies [33] which showed significant losses of tocopherols in oil during heating. δ-tocopherol was lost much more slowly than other isomers, particularly α-tocopherols. Simultaneously, these data are in contradiction with results obtained by other authors, who noticed a more advanced decomposition of γ-tocopherol than α-tocopherol [34]. In turn, Aladedunye & Przybylski [35] pointed out that the relative loss of tocopherol isomers was γ > α > δ and γ > δ > α in two different high-oleic sunflower oils. Further, Aladedunye & Przybylski [35] stated after Warner & Moser [36] that a tocopherol isomer with higher content degrades much faster than another isomer with lower content. This pattern generally corresponds to findings in the present study, except for γ-tocopherol which was less susceptible to degradation than α-tocopherol; γ-tocopherol was, however, characterized with the highest level among tocochromanols. The above variances in results may be the effect of different investigation conditions, because the aforementioned researchers carried out the deep-frying of food like frozen French fries for a few hours or several days rather than heating the oil on the pan in a thin layer for short time.

#### 2.2.2. Total Polar Compounds (TPC)

**Figure 2:**
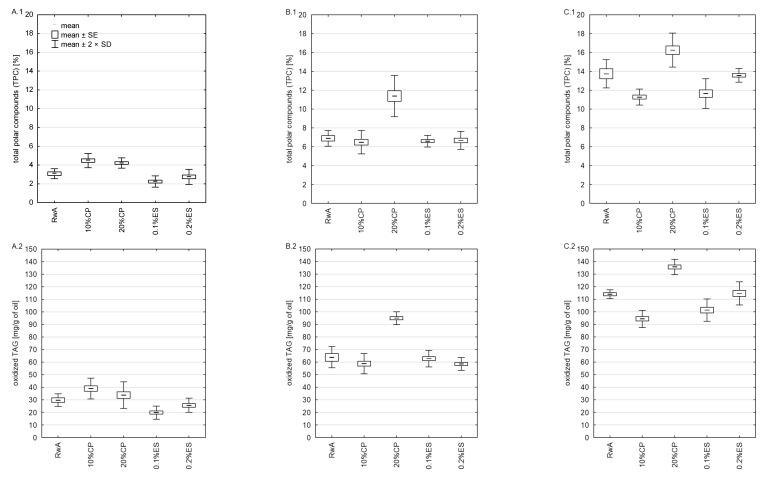

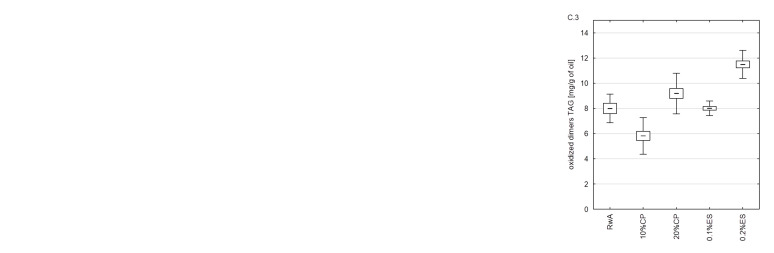
Products of thermal degradation of oils. Values are means of four determinations. (**A.1**,**A.2**)—not heated samples; (**B.1**,**B.2**)—samples heated at 170 °C; (**C.1**–**C.3**)—samples heated at 200 °C; SE—standard error; SD—standard deviation.

The TPC results of the nonheated samples and the samples heated at 170 and 200 °C are presented in Figure 2. In this research, TPC in nonheated samples ranged from 2.25 (0.1% ES) to 4.48% (20% CP). There were no significant (*p* < 0.05) differences between 0.1% ES, 0.2% ES, and RwA; however, these samples were considerably different from 10% CP and 20% CP.

The heating process caused an increase in TPC level and ranged from 6.48% (0.1% ES) to 11.33% (20% CP) in samples heated at 170 °C. There were no significant (*p* < 0.05) differences in the TPC content between the samples heated at 170 °C, except for the 20% CP sample which achieved an almost twice-higher value compared to the other sample. These results might relate to the highest PUFA share and the lowest tocochromanol initial total content in 20% CP, which differed noticeably from the initial tocochromanol level in other samples.

Among the samples heated at 200 °C, the difference between the 10% CP and 20% CP samples is noteworthy. Similar to 170 °C, the 20% CP sample distinguished itself from others by the highest TPC content (16.24%). On the other hand, the lowest value of this parameter was found in the 10% CP (11.26%) sample. This was observed even though 10% CP contained 2.86% more PUFA than RwA and only 3.5% less than 20% CP. In another study [37], a blend of sunflower oil (80%) and Moringa oleifera oil (20%) was used during deep-frying of fresh potato cubes at 180 °C. The blend showed a lower TPC level than sunflower oil. This can be attributed to the presence of minor compounds in moringa oleifera oil such as sterols or vitamin E, but the fatty acid composition should also be noted. Moringa oleifera oil was characterized by high MUFA content; in turn, sunflower oil was demonstrated to have high PUFA content. Moreover, when Kiralan et al. [38] researched blends of sunflower oil and black cumin oil, they found better oxidative stability in blends than in sunflower oil, and this was explained by the effect of thymoquinone and tocopherols. In the present work, it can be assumed that the bioactive substances contained in the 10% CP oil sample had a positive effect on reducing TPC formation despite the increased PUFA content compared to RwA. On the other hand, in the 20% CP sample, the effect of increased PUFA content turned out to be dominant, which resulted in greater PUFA susceptibility to thermal degradation and more TPC production under the influence of heating. Erkan et al. [39] showed that the addition of black cumin essential oil improved the stabilization of sunflower oil under accelerated storage conditions; however, methanol extract of rosemary turned out to be more effective. To the best of our knowledge, black cumin essential oil was never previously researched for retarded oil degradation during frying. Nevertheless, essential oils of plants such as black pepper, ginger [40], laurel, oregano, and rosemary [41] were studied under similar conditions, and they exhibited great protective potential during frying. It was demonstrated that essential oils containing carvacrol or its derivatives are effective in inhibiting oil deterioration, and this can be attributed to their polyphenol structure [41,42]. However, other compounds in essential oils without a polyphenol structure can also show a protective effect during frying. Limonene, one of the nutmeg essential oils [43], and mentha spicata essential oil [14] components were considered to significantly increase the oxidative stability of sunflower oil during the deep-frying process.

#### 2.2.3. TAG Polymers Composition and Content

Oxidized TAG content (Figure 2) increased gradually, starting with unheated oils, through oils heated at 170 °C, and ending with oils heated at 200 °C. Dimers TAG were found only in samples heated at 200 °C (Figure 2); in turn, trimers and oligomers were not detected in any samples. These parameter values corresponded with TPC because oxidized TAG are the predominant component of polar compounds. Oxidized TAG content in nonheated samples ranged from 19.85 (0.1% ES) to 38.97 mg·g oil^−1^ (10% CP). There was no difference in protective effect between samples heated at 170 °C (58.48–63.74 mg·g oil^−1^); only the 20% CP sample had a significantly (*p* < 0.05) higher level (94.85 mg·g oil^−1^). Simultaneously, a statistically significant (*p* < 0.05) protective effect in decreasing oxidized TAG content at 200 °C was observed for 10% CP, which reached the lowest level (94.88 mg·g oil^−1^). However, this effect was not observed for the other samples; indeed, the higher concentration of cold-pressed increased oxidized TAG content. Moreover, 10% CP showed the lowest dimers content (5.81 mg·g oil^−1^) and differed significantly (*p* < 0.05) from other samples. According to our previous investigation, the addition of a tea-and-fruits extract [15] or, based on other authors’ study, camellia oleifera seed cake extract [44] can significantly reduce the polymers level in frying oil. Nevertheless, the low solubility of these additives limits prominently the possibility of their practical application. Next, research showed that fat-soluble substances like nutmeg [43], mentha spicata [14], and gardenia jasminoides fruits [42] essential oils exhibited considerable anti-polymerization activity, and it can be assumed that this is the consequence of the oxidative stability effect of their components (limonene or carvacrol methyl ether). Furthermore, Rodríguez, et al. [45] deep-frying sacha inchi cold-pressed oil had a significantly lower polymerization level than soybean oil, despite a high PUFA level (α-linolenic acid 53.8% and linoleic acid 33.4%). This was related to high tocopherol content and proved that sacha inchi cold-pressed oil is suitable for short-term deep-frying.

## 3. Principal Component Analysis (PCA)

The results of PCA indicated that the first two principal components (PC1 × PC2) explain 90.7% of the variance in the original data.

**Figure 3:**
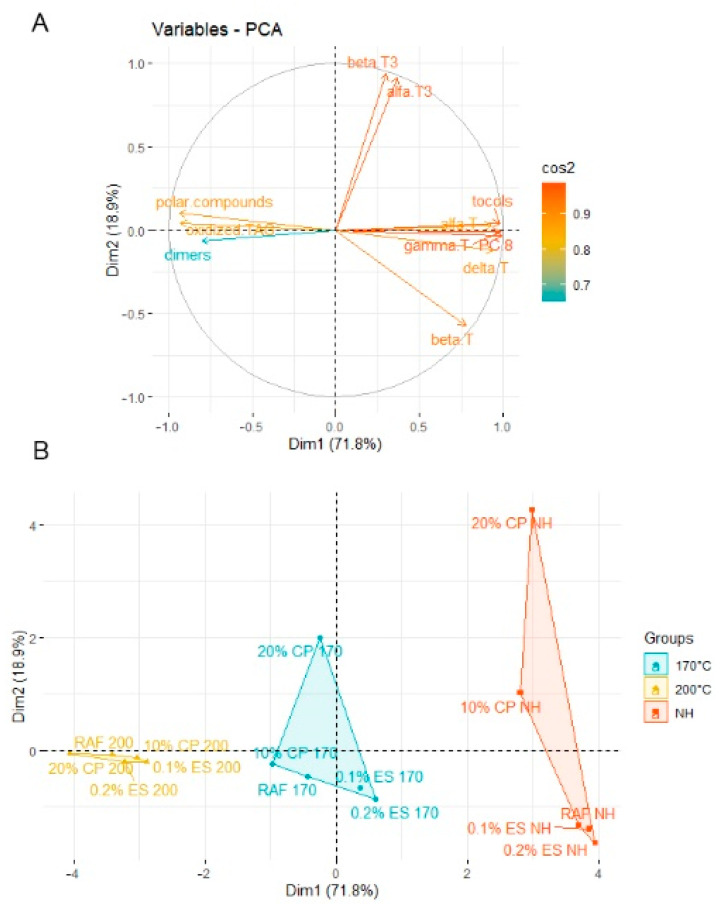
The changes Score plot (**A**) and loading plot (**B**) of principal component analysis (PCA) for data from α-, β-, γ-, and δ-tocopherols (T), plastochromanol-8 (PC-8), tocochromanols (tocol), total polar compounds (TPC), oxidized triacylglycerol (oxidized TAG), and dimers triacylglycerol (dimers TAG).

As showed in Figure 3A, PC1 (71.8% of the total variance) was positively correlated with total tocopherols (0.980972), α-tocopherol (0.930), β-tocopherol (0.776), γ-tocopherol (0.987), δ-tocopherol (0.943), and PC-8 (0.993) content. Subsequently, it was negatively correlated with TPC (−0.946), oxidized TAG (−0.955), and dimers (−0.819) content. In turn, PC2 (18.9% of the total variance) was positively correlated with α- tocotrienol (0.919) and β-tocotrienol (0.938) content.

Most tested samples were distributed along with factor 1 axis into three major clusters (Figure 3B), except for the three samples CP10% 0 °C, CP20% 0 °C, and CP20% 170 °C, which were classified as outliers. Cluster 1 (red area) contained three nonheated samples with high total tocopherols content and low TPC and dimers content, related to the positive zone of the F1-axis. Cluster 2 (blue area) included four samples heated at 170 °C and characterized by lower total tocopherols content and higher TPC and dimers content, in the middle zone of the F1-axis. Finally, cluster 3 (yellow area) embraced five samples heated at 200 °C which were the most degraded, associated with a negative zone of the F1-axis.

## 4. Materials and Methods

### 4.1. Research Material

Refined rapeseed oil without additives (RwA) was purchased at a grocery store, and cold-pressed black cumin oil (CP) was delivered directly from an oil manufacturer.

### 4.2. Preparation and Composition Analysis of Black Cumin Essential Oil

In this investigation, black cumin essential oil (ES) was prepared by the hydrodistillation of cold-pressed black cumin oil (CP) using a Clevenger-type apparatus and analyzed by gas chromatography combined with mass spectrometry according to previous methodology [46]. Essential oil composition was identified by comparison to mass spectrum libraries (NIST05), by using oil cannabis standard, and by calculating Kovats retention indices (RI) based on homologous series of n-alkanes C9–C14.

### 4.3. Preparation of Fortified Oils

Four fortified oils were prepared. Two of them were blends of cold-pressed black cumin oils. They were prepared by adding 10 and 20% (*v*/*v*) of cold-pressed oil to refined rapeseed oil (10% CP and 20% CP samples, respectively). The next two fortified oils were mixtures of black cumin essential oil. The essential oil yield from black cumin seed oil was 1%. Therefore, to obtain a comparable content of essential oil in blends with cold-pressed black cumin oil and mixtures with essential oil, mixtures with essential oil were prepared by adding 0.1 and 0.2% (*v*/*v*) of essential oil to refined rapeseed oil (0.1% ES and 0.2% ES samples, respectively), which corresponded to an addition of 10% and 20% cold-pressed black cumin oil. Then the solution was mixed by shaking for 5 min at room temperature. All fortified oils were stored under nitrogen, in the dark, at −30 °C until the analysis.

### 4.4. Heating Procedure

**Figure 4:**
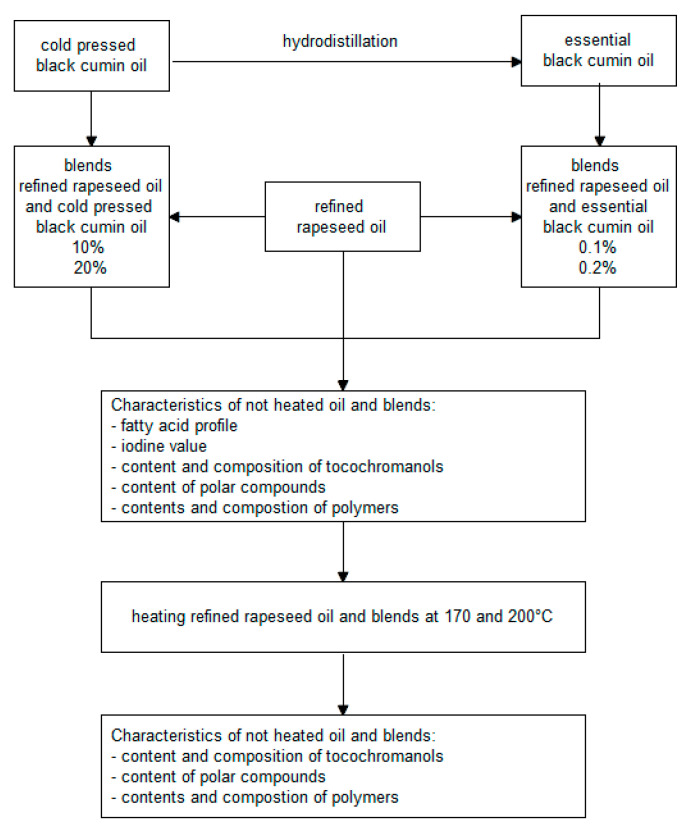
Graphical scheme of study approach.

Each sample of fortified oil and refined rapeseed oil was heated at two temperatures: 170 and 200 °C. For both temperatures, the process was carried out on a pan with a diameter of 20 cm and performed in two repetitions. 50 mL of oil was heated in a thin layer, by hotplates MS-H-Pro (Scilogex, Rocky Hill, CT, USA) with temperature control. In the first stage, oils were heated for 7 min to reach 170 °C or 9 min to 200 °C. In the second stage, oils were heated for 10 min, maintaining a temperature of 170 or 200 °C. The actual pan oil temperature was verified using an electronic thermometer testo 104 (Testo Sp. z o. o., Pruszków, Poland). After chilling, the oils samples were transferred to plastic containers in which the remaining free space was filled with nitrogen. Closed sample containers were stored under nitrogen, without access to light and at a temperature −30 °C until the analysis. The manufacturing stages of fortified oil and the analytical procedure are presented in Figure 4.

### 4.5. Fatty Acid Composition

The fatty acid composition was determined according to the AOCS Official Method Ce 1h-05 [47]. Oil samples were dissolved in hexane and transesterified with sodium methylate. Fatty acid methyl esters (FAME) were analyzed with an Agilent 7820A GC (Agilent Technologies, Santa Clara, CA, USA) equipped with a flame ionization detector (FID) and a SLB-IL111 capillary column (Supelco, Bellefonte, PA, USA) (100 m, 0.25 mm, 0.20 μm). FAME was separated under the following conditions: the initial oven temperature was 150 °C and it was increased to 200 °C at 1.5 °C/min; the injector and detector temperature was 250 °C; the split was 1:10; and the carrier gas was helium at 1 mL/min. The fatty acid methyl esters (FAME) were identified by comparison with commercially available standards: grain fatty acid methyl ester mix (Supelco, Bellefonte, PA, USA). The results were expressed as a percentage of total fatty acids.

### 4.6. Iodine Value Calculation

The iodine value is the measure of the degree of unsaturation of fat. It was calculated according to the AOCS Official Method Cd 1c-85 [48]. It is based on percentage of hexadecenoic acid, octadecenoic acid, octadecadienoic acid, octadecatrienoic acid, eicosanoid acid, and docosenoic acid.

### 4.7. Tocochromanols Analysis

The total tocochromanol content and composition were determined according to Siger et al. [49]. The oil was dissolved in n-hexane and transferred to vials for analysis. The tocochromanol content was analyzed using Waters HPLC system (Waters, Milford, MA, USA) equipped with a LiChrosorb Si 60 column (Merck, Darmstadt, Germany) (250 mm, 4.6 mm, 5 μm), a fluorimetric detector (Waters 474), and a photodiode array detector (Waters 2998 PDA). The mobile phase was a mixture of n-hexane with 1.4-dioxane (96:4 *v*/*v*). The flow rate was 1.0 mL/min (for tocochromanols). To detect the fluorescence of tocochromanols, the excitation wavelength was set at ʎ = 295 nm and the emission wavelength at ʎ = 330 nm. Standards of all tocochromanols (>95% of purity) were purchased from Merck (Darmstadt, Germany).

### 4.8. Total Polar Compounds (TPC) Analysis

The content of polar compounds in the oils was analyzed according to AOCS Official Method 982.27 (1984) [50]. Concisely, the oil sample was divided into a nonpolar and a polar fraction using silica gel columns. The nonpolar fraction was eluted with a mixture of hexane and diisopropyl ether (82:18, *v*/*v*). Then the collected nonpolar fraction was treated by evaporation to remove the solvent and weighed. Next, the polar fraction was calculated based on the weight difference of the sample and the nonpolar fraction. The results were expressed as a percentage of the total content of the oil sample.

### 4.9. Triacylglycerols (TAG) Polymers Analysis

Dimers, trimers, and oligomers of TAG were evaluated in the polar fraction according to Kmiecik et al. [15]. The analysis was conducted by high-performance size-exclusion chromatography (HPSEC) using Infinity 1290 HPLC (Agilent Technologies, Santa Clara, CA, USA) coupled with ELSD (Evaporative Light Scattering Detector) and two connected Phenogel columns (100 Å and [24] 500 Å, 5 μ, 300 × 7.8 mm) (Phenomenex, Torrance, CA, USA). The liquid phase was dichloromethane (DCM).

### 4.10. Statistical Analysis

All tests were run in duplicate. The findings of fatty acids and tocochromanols in nonheated oils were expressed as means ± standard deviation. Analysis of variance (ANOVA) and Tukey’s post hoc tests were used to determine statistically significant values (*p* ≤ 0.05) between these samples. The averaged findings of tocochromanols, TPC, content of oxidized TAG, and dimers TAG in heated oil were counted based on two repetitions of heating. Box-and-whisker plots presented the results of polar content and polymer composition, where the mean is denoted with a horizontal line in the box, standard error (SE) is between the top and bottom of the box, and standard deviation (SD) is shown by the ends of the whiskers. Principal component analysis (PCA) was performed for the results of total polar component, content oxidized TAG, total tocochromanols, the content of α- β- γ- δ tocopherol, and PC-8. STATISTICA software (version 13.3, StatSoft, Tulsa, OK, USA) and R software (version 4.1 with packages FactoMineR v.2.4 and factoextra v.1.0.7) were used for the data analysis.

## 5. Conclusions

Initial tocopherols content in all the samples did not differ noticeably, and this is related to the similar TPC and dimers levels between the samples heated at 170 °C. The exception is the CP20% sample, which achieved a higher content of TPC and dimers; this could have resulted from the lowest initial tocopherols content as well as from the high PUFA share that turned out to be dominant in this sample.

However, a much more advanced decline in tocochromanols was demonstrated at 200 °C, and for this reason the tocochromanols ceased to exhibit protective activity, whichleded to a successive increase in TPC and dimers content. Only the addition of 10% black cumin oil reduced the TPC and dimers formation at 200 °C, and this can be attributed to the activity of other compounds, such as sterols or polyphenols; but this hypothesis should be verified and researched more precisely in further studies. The above results lead to the conclusion that using refined rapeseed oil and cold-pressed black cumin seed oil blend (90:10% *v*/*v*) during heating at 200 °C temperature gives more benefits than using refined rapeseed oil without additives. Simultaneously, in CP20%, prooxidant PUFA properties were still outbalanced over other factors, and this sample touched the highest degradation level.

The addition of black cumin essential oil contributed no diminishment in TPC and dimers content. These results indicated that the main component of essential oil, thymoquinone, displayed no protective effect against oils during heating or was destroyed very quickly in the high temperature of the process. Nevertheless, black cumin essential oil with a different composition—for example, a composition containing carvacrol, thymol, or its derivatives—could have more beneficial properties; however, this situation deserves to be further explored.

Another issue that needs to be examined in following studies is the sensory evaluation of fortified oils and blends in terms of consumer preferences.

## Figures and Tables

**Table 1:** Chemical composition of essential black cumin oil.

No.	Compounds	Concentration (%)	Retention Time (min)	RI
1	α-thujene	15.32	7.811	933
2	α-pinene	3.94	7.99	943
3	sabinene	1.60	8.729	980
4	β-pinene	3.43	8.866	987
5	3-Carene	0.81	9.561	1024
6	p-cymene	33.51	9.709	1033
7	limonene	4.08	9.799	1038
8	γ-terpinene	4.37	10.317	1066
9	cis-4-methoxythujane	0.97	11.011	1102
10	trans-4-methoxythujane	4.76	11.429	1128
11	terpinen-4-ol	0.39	12.563	1192
12	β-cyclocitral	0.73	12.863	1210
13	thymoquinone	23.55	13.646	1260
14	α-longipinene	0.60	15.405	1373
15	longifolene	1.93	16.363	-

**Table 2:** Fatty acid composition and iodine value in nonheated oils.

	RwA	10% CP	20% CP	0.1% ES	0.2% ES
[%]					
16:0	4.52 ± 0.08 c	5.44 ± 0.16 b	6.04 ± 0.14 a	4.63 ± 0.00 c	4.60 ± 0.13 c
16:1	0.17 ± 0.01 a	0.18 ± 0.01 a	0.18 ± 0.00 a	0.18 ± 0.01 a	0.18 ± 0.01 a
18:0	1.74 ± 0.03 c	1.83 ± 0.00 b	1.96 ± 0.00 a	1.71 ± 0.01 c	1.72 ± 0.00 c
18:1	65.60 ± 0.14 a	61.6 ± 0.06 b	57.69 ± 0.28 c	65.22 ± 0.03 a	65.18 ± 0.02 a
18:2	18.46 ± 0.06 a	22.04 ± 0.06 b	25.91 ± 0.27 c	18.49 ± 0.01 a	18.52 ± 0.06 a
20:0	0.66 ± 0.01 a	0.68 ± 0.02 b	0.63 ± 0.01 b	0.75 ± 0.00 a	0.74 ± 0.00 a
18:3	8.39 ± 0.03 a	7.80 ± 0.13 b	7.18 ± 0.12 c	8.53 ± 0.01 a	8.57 ± 0.05 a
22:0	0.29 ± 0.01 a	0.26 ± 0.02 ab	0.24 ± 0.01 b	0.30 ± 0.00 a	0.30 ± 0.01 a
22:1	0.17 ± 0.01 b	0.18 ± 0.00 ab	0.16 ± 0.00 b	0.20 ± 0.00 a	0.20 ± 0.00 a
SFA	7.22 ± 0.03 c	8.21 ± 0.12 b	8.87 ± 0.12 a	7.39 ± 0.01 c	7.36 ± 0.13 c
MUFA	65.94 ± 0.12 a	61.96 ± 0.07 b	58.03 ± 0.27 c	65.6 ± 0.01 a	65.55 ± 0.01 a
PUFA	26.84 ± 0.09 c	29.70 ± 0.18 b	33.20 ± 0.15 a	27.02 ± 0 c	27.17 ± 0.11 c
Iodine value	110.61 ± 0.08 c	111.84 ± 0.37 b	113.57 ± 0.07 a	110.75 ± 0.00 c	110.86 ± 0.25 c

Values are means of two determinations ± SD. Means in the same row followed by different letters indicate significant differences (*p* < 0.05) between samples. RwA—refined rapeseed oil without addition; 10% CP—refined rapeseed oil with addition of 10% cold-pressed oil; 20% CP—refined rapeseed oil with addition of 20% cold-pressed oil; 0.1% ES—refined rapeseed oil with addition of 0.1% essential oil; 0.2% ES—refined rapeseed oil with addition of 0.2% essential oil; SFA—saturated fatty acid; MUFA—monounsaturated fatty acids; PUFA—polyunsaturated fatty acids.

**Table 3:** The content of tocochromanols and their homologues in nonheated oils.

	RwA	10% CP	20% CP	0.1% ES	0.2% ES
[mg/100 g]					
α-tocopherol	28.71 ± 0.45 a	25.82 ± 0.36 ab	23.78 ± 0.60 b	28.69 ± 1.24 a	29.26 ± 0.75 b
β-tocopherol	0.12 ± 0.03 a	0.05 ± 0.01 a	0.05 ± 0.01 a	0.13 ± 0.03 a	0.15 ± 0.03 a
γ-tocopherol	32.39 ± 0.85 ba	30.08 ± 0.51 b	27.67 ± 0.63 c	32.21 ± 0.27 ba	33.65 ± 0.81 a
δ-tocopherol	0.73 ± 0.05 a	0.56 ± 0.01 bc	0.54 ± 0.04 c	0.65 ± 0.02 ab	0.67 ± 0.04 ab
α-tocotrienol	nd	0.55 ± 0.05 b	1.12 ± 0.08 a	nd	nd
β-tocotrienol	nd	0.78 ± 0.05 b	2.72 ± 0.11 a	nd	nd
PC-8	4.55 ± 0.43 a	4.22 ± 0.06 a	3.81 ± 0.23 a	4.53 ± 0.23 a	4.62 ± 0.06 a
Total tocochromanols	66.49 ± 1.70 a	62.06 ± 0.95 b	59.65 ± 1.17 b	66.21 ± 1.64 a	68.35 ± 1.63 a

Values are means of two determinations ± SD. Means in the same row followed by different letters indicate significant differences (*p* < 0.05) between samples. RwA—refined rapeseed oil without addition; 10% CP—refined rapeseed oil with addition of 10% cold-pressed oil; 20% CP—refined rapeseed oil with addition of 20% cold-pressed oil; 0.1% ES—refined rapeseed oil with addition of 0.1% essential oil; 0.2% ES—refined rapeseed oil with addition of 0.2% essential oil; PC-8—plastochromanol-8; nd—not detected.

## Data Availability

Not applicable.

## Sample Availability

Samples of the compounds are not available from the authors.

## References

[1] Perez-Herrera A., Rangel-Zuñiga O.A., Delgado-Lista J., Marin C., Perez-Martinez P., Tasset I., Tunez I., Quintana-Navarro G.M., Lopez-Segura F., De Castro M.D.L. (2013). The antioxidants in oils heated at frying temperature, whether natural or added, could protect against postprandial oxidative stress in obese people. Food Chem..

[2] Hosseini H., Ghorbani M., Meshginfar N., Mahoonak A.S. (2016). A Review on Frying: Procedure, Fat, Deterioration Progress and Health Hazards. JAOCS J. Am. Oil Chem. Soc..

[3] Cao W., Wang X., Zhang W., Wang X. (2013). Toxic effects of triacylglycerol polymer on macrophages in vitro. Eur. J. Lipid Sci. Technol..

[4] Li X., Yu X., Sun D., Li J., Wang Y., Cao P., Liu Y. (2017). Effects of polar compounds generated from the deep-frying process of palm oil on lipid metabolism and glucose tolerance in kunming mice. J. Agric. Food Chem..

[5] López-Varela S., Sánchez-Muniz F.J., Cuesta C. (1995). Decreased food efficiency ratio, growth retardation and changes in liver fatty acid composition in rats consuming thermally oxidized and polymerized sunflower oil used for frying. Food Chem. Toxicol..

[6] Sánchez-Muniz F.J., López-Varela S., Garrido-Polonio M.C., Cuesta C. (1998). Dietary effects on growth, liver peroxides, and serum and lipoprotein lipids in rats fed a thermoxidised and polymerised sunflower oil. J. Sci. Food Agric..

[7] Johnson O.C., Kummerow F.A. (1957). Chemical changes which take place in an edible oil during thermal oxidation. J. Am. Oil Chem. Soc..

[8] Santos J.C.O., Santos I.M.G., Souza A.G. (2005). Effect of heating and cooling on rheological parameters of edible vegetable oils. J. Food Eng..

[9] Dobarganes C., Márquez-Ruiz G., Velasco J. (2000). Interactions between fat and food during deep-frying. Eur. J. Lipid Sci. Technol..

[10] Cahill L.E., Pan A., Chiuve S.E., Sun Q., Willett W.C., Hu F.B., Rimm E.B. (2014). Fried-food consumption and risk of type 2 diabetes and coronary artery disease: A prospective study in 2 cohorts of US women and men. Am. J. Clin. Nutr..

[11] Sayon-Orea C., Bes-Rastrollo M., Basterra-Gortari F.J., Beunza J.J., Guallar-Castillon P., de la Fuente-Arrillaga C., Martinez-Gonzalez M.A. (2013). Consumption of fried foods and weight gain in a Mediterranean cohort: The SUN project. Nutr. Metab. Cardiovasc. Dis..

[12] Sayon-Orea C., Martinez-Gonzalez M.A., Gea A., Flores-Gomez E., Basterra-Gortari F.J., Bes-Rastrollo M. (2014). Consumption of fried foods and risk of metabolic syndrome: The SUN cohort study. Clin. Nutr..

[13] Gadiraju T.V., Patel Y., Gaziano J.M., Djoussé L. (2015). Fried food consumption and cardiovascular health: A review of current evidence. Nutrients.

[14] Wang D., Chen X., Wang Q., Meng Y., Wang D., Wang X. (2020). Influence of the essential oil of Mentha spicata cv. Henanshixiang on sunflower oil during the deep-frying of Chinese Maye. LWT.

[15] Kmiecik D., Gramza-Michałowska A., Korczak J. (2018). Anti-polymerization activity of tea and fruits extracts during rapeseed oil heating. Food Chem..

[16] Kostadinović Veličkovska S., Brühl L., Mitrev S., Mirhosseini H., Matthäus B. (2015). Quality evaluation of cold-pressed edible oils from Macedonia. Eur. J. Lipid Sci. Technol..

[17] Bozdoğan Konuşkan D. (2020). Minor bioactive lipids in cold pressed oils. Cold Pressed Oils.

[18] Durazzo A., Fawzy Ramadan M., Lucarini M. (2022). Editorial: Cold Pressed Oils: A Green Source of Specialty Oils. Front. Nutr..

[19] Ketenoglu O., Kiralan S.S., Kiralan M., Ozkan G., Ramadan M.F. (2020). Cold pressed black cumin (Nigella sativa L.) seed oil. Cold Pressed Oils.

[20] Mazaheri Y., Torbati M., Azadmard-Damirchi S., Savage G.P. (2019). A comprehensive review of the physicochemical, quality and nutritional properties of *Nigella sativa* oil. Food Rev. Int..

[21] Kazemi M. (2014). Phytochemical Composition, Antioxidant, Anti-inflammatory and Antimicrobial Activity of *Nigella sativa* L. Essential Oil. J. Essent. Oil-Bear. Plants.

[22] Lutterodt H., Luther M., Slavin M., Yin J., Parry J., Gao J., Lucy L. (2010). Fatty acid profile, thymoquinone content, oxidative stability, and antioxidant properties of cold-pressed black cumin seed oils. LWT—Food Sci. Technol..

[23] Erkan N., Ayranci G., Ayranci E. (2008). Antioxidant activities of rosemary (*Rosmarinus Officinalis* L.) extract, blackseed (*Nigella sativa* L.) essential oil, carnosic acid, rosmarinic acid and sesamol. Food Chem..

[24] Kokoska L., Havlik J., Valterova I., Sovova H., Sajfrtova M., Jankovska I. (2008). Comparison of chemical composition and antibacterial activity of Nigella sativa seed essential oils obtained by different extraction methods. J. Food Prot..

[25] Burits M., Bucar F. (2000). Antioxidant activity of Nigella sativa essential oil. Phyther. Res..

[26] Jrah Harzallah H., Kouidhi B., Flamini G., Bakhrouf A., Mahjoub T. (2011). Chemical composition, antimicrobial potential against cariogenic bacteria and cytotoxic activity of Tunisian *Nigella sativa* essential oil and thymoquinone. Food Chem..

[27] Khalid A.K., Shedeed M.R. (2016). Yield and Chemical Composition of Nigella sativa L. Essential Oil Produced under Kinetin Treatments. J. Essent. Oil-Bear. Plants.

[28] Konuskan D.B., Arslan M., Oksuz A. (2019). Physicochemical properties of cold pressed sunflower, peanut, rapeseed, mustard and olive oils grown in the Eastern Mediterranean region. Saudi J. Biol. Sci..

[29] Kiralan M., Özkan G., Bayrak A., Ramadan M.F. (2014). Physicochemical properties and stability of black cumin (*Nigella sativa*) seed oil as affected by different extraction methods. Ind. Crops Prod..

[30] Ramadan M.F. (2013). Healthy blends of high linoleic sunflower oil with selected cold pressed oils: Functionality, stability and antioxidative characteristics. Ind. Crops Prod..

[31] Schwartz H., Ollilainen V., Piironen V., Lampi A.M. (2008). Tocopherol, tocotrienol and plant sterol contents of vegetable oils and industrial fats. J. Food Compos. Anal..

[32] Kasprzak M., Rudzińska M., Przybylski R., Kmiecik D., Siger A., Olejnik A. (2020). The degradation of bioactive compounds and formation of their oxidation derivatives in refined rapeseed oil during heating in model system. LWT.

[33] Saoudi S., Chammem N., Sifaoui I., Bouassida-Beji M., Jiménez I.A., Bazzocchi I.L., Silva S.D., Hamdi M., Bronze M.R. (2016). Influence of Tunisian aromatic plants on the prevention of oxidation in soybean oil under heating and frying conditions. Food Chem..

[34] Matthäus B. (2006). Utilization of high-oleic rapeseed oil for deep-fat frying of French fries compared to other commonly used edible oils. Eur. J. Lipid Sci. Technol..

[35] Aladedunye F., Przybylski R. (2013). Frying stability of high oleic sunflower oils as affected by composition of tocopherol isomers and linoleic acid content. Food Chem..

[36] Warner K., Moser J. (2009). Frying stability of purified mid-oleic sunflower oil triacylglycerols with added pure tocopherols and tocopherol mixtures. JAOCS J. Am. Oil Chem. Soc..

[37] Boukandoul S., Santos C.S.P., Casal S., Zaidi F. (2019). Oxidation delay of sunflower oil under frying by moringa oil addition: More than just a blend. J. Sci. Food Agric..

[38] Kiralan M., Ulaş M., Özaydin A., Özdemır N., Özkan G., Bayrak A., Ramadan M.F. (2017). Blends of Cold Pressed Black Cumin Oil and Sunflower Oil with Improved Stability: A Study Based on Changes in the Levels of Volatiles, Tocopherols and Thymoquinone during Accelerated Oxidation Conditions. J. Food Biochem..

[39] Erkan N., Ayranci G., Ayranci E. (2012). Lipid oxidation inhibiting capacities of black seed essential oil and rosemary extract. Eur. J. Lipid Sci. Technol..

[40] Chandran J., Nayana N., Roshini N., Nisha P. (2017). Oxidative stability, thermal stability and acceptability of coconut oil flavored with essential oils from black pepper and ginger. J. Food Sci. Technol..

[41] Olmedo R.H., Asensio C.M., Grosso N.R. (2015). Thermal stability and antioxidant activity of essential oils from aromatic plants farmed in Argentina. Ind. Crops Prod..

[42] Wang D., Wang Q., Li S., Xu Y., Wang X., Wang C. (2020). Carvacrol methyl ether, a compound from the essential oil of Gardenia jasminoides fruits, exhibits antioxidant effects in the deep-frying of Chinese Youmotou using sunflower oil. LWT.

[43] Wang D., Dong Y., Wang Q., Wang X., Fan W. (2020). Limonene, the compound in essential oil of nutmeg displayed antioxidant effect in sunflower oil during the deep-frying of Chinese Maye. Food Sci. Nutr..

[44] Wu G., Han S., Zhang Y., Liu T.T., Karrar E., Jin Q., Zhang H., Wang X. (2022). Effect of phenolic extracts from Camellia oleifera seed cake on the formation of polar compounds, core aldehydes, and monoepoxy oleic acids during deep-fat frying. Food Chem..

[45] Rodríguez G., Squeo G., Estivi L., Quezada Berru S., Buleje D., Caponio F., Brandolini A., Hidalgo A. (2021). Changes in stability, tocopherols, fatty acids and antioxidant capacity of sacha inchi (*Plukenetia volubilis*) oil during French fries deep-frying. Food Chem..

[46] Myszka K., Schmidt M.T., Majcher M., Juzwa W., Czaczyk K. (2017). β-Caryophyllene-rich pepper essential oils suppress spoilage activity of Pseudomonas fluorescens KM06 in fresh-cut lettuce. LWT—Food Sci. Technol..

[47] AOCS (2005). AOCS Official Method Ce 1h-05. Determination of cis-, trans-, Saturated, Monounsaturated and Polyunsaturated Fatty Acids in Vegetable or Non-ruminant Animal Oils and Fats by Capillary GLC. Official Methods and Recommended Practices of the American Oil Chemists’ Society.

[48] AOCS (2009). AOCS Official Method Cd 1c-85. Calculated iodine value. Official Methods and Recommended Practices of the American Oil Chemists’ Society.

[49] Siger A., Michalak M., Rudzińska M. (2016). Canolol, tocopherols, plastochromanol-8, and phytosterols content in residual oil extracted from rapeseed expeller cake obtained from roasted seed. Eur. J. Lipid Sci. Technol..

[50] AOCS (1984). AOCS Official Method 982.27. Polar components in frying fats. Official Methods and Recommended Practices of the American Oil Chemists’ Society.

